# Circulating Tumor Cells Detection in Patients with Early Breast Cancer Using MACS Immunomagnetic Flow Cytometry

**Published:** 2020

**Authors:** Nasrin Karimi, Mana Oloomi, Zahra Orafa

**Affiliations:** Department of Molecular Biology, Pasteur Institute of Iran, Tehran, Iran

**Keywords:** Circulating tumor cells, Cytokeratins, EpCAM, Epithelial cell adhesion molecule, Flow cytometry

## Abstract

**Background:**

Circulating Tumor Cells (CTCs) detection in peripheral blood of epithelial cancer patients is an indicator of the presence of primary tumors and metastasis. The CTC phenotype detection uses epithelial markers in defining, detecting, and isolating CTCs. Circulating cell-separation technologies, with the epithelial origin, can be identified by epithelial biomarkers, with different techniques such as flow cytometry. The purpose of this study was to evaluate the expression of molecular Cytokeratins (CKs), CK7, CK8, CK18, CK19 (Pan-CK) and Epithelial Cell Adhesion Molecule (EpCAM) markers for CTC detection.

**Methods:**

The Magnetic Activated Cell Sorting (MACS) was used to identify CTCs in the blood of patients. Specific antibodies to EpCAM and Pan-CK were used and analyzed by flow cytometry. In this study, 35 blood samples of patients with breast cancer were assessed before any treatment and 35 healthy blood samples as the control were evaluated.

**Results:**

Expression of CK markers in the peripheral blood of breast cancer patients was statistically significant with p≤0.05, specifically at stages II-IV, but it was not significant in patients at stage I and healthy controls. Biomarkers expression in the blood of patients and healthy controls was assessed along with the pathologic characteristics of patients.

**Conclusion:**

CTC assessment by flow cytometry in patients with breast cancer could not only be used for detection but also can be considered as a source of specific and subjective evaluation for monitoring the therapy. Besides, the sensitivity and specificity of CTC detection were shown that could be enhanced by specific CK markers.

## Introduction

Breast cancer is the oldest diagnosed cancer ^1^, which is more seen in women and is the most common deadly cancer ^2,3^. It is considered the second cause of cancer death in women after lung cancer in Iran ^4^. According to national reports of cancer cases, breast cancer is the first universal kind of cancer among Iranian women and accounts for 16% of all cancers ^3^. The current treatments of breast cancer include the surgery and chemotherapy, and there is the possibility of disease recurrence in most of the cases due to the presence of Circulating Tumor Cells (CTCs) ^5^. CTCs are tumor cells which can be detected in the blood of patients with different types of early to advance breast cancer. It is very heterogeneous with significant clinical implycations. In breast cancer, these cells are a rare population of cells in the blood and have an essential role in the early diagnosis of cancer development. Firstly, Thomas Ashworth diagnosed these cells in 1867 ^6^. As it is mentioned, CTCs are highly heterogeneous with critical molecular properties ^7^. It seems that they are trapped in organs in the first few minutes of entering the blood, and their presence in the blood is very short ^8^. Breast cancer is among the diseases with epithelial origins, and its specific tumor markers express on these epithelial cells ^5^. There are few studies conducted on the diagnosis of CTC at the early stages of breast cancer ^9^. Recently, assessment of CTCs has been shown for early detection of metastatic breast cancer and monitoring of treatment response in breast cancer ^10^. CTCs contain prognostic information in metastatic breast cancer patients ^11^.

Different markers are used to diagnose the CTC, including the epithelial markers such as Cytokeratins (CKs), and also the Epithelial Cell Adhesion Molecule (EpCAM) ^12^. CKs are the large protein structures in epithelial cells from the family of intermediate filaments ^13^. More than 20 different types of CKs including CK8, CK18, and CK19 as the most abundant CKs of epithelial cells are involved in cancers such as breast, prostate, lung, and colon cancer ^14^. Several research groups have reported that CK8 and CK18 have a simultaneous expression in a variety of tumors ^15^. CK18 with CK8 also express in a variety of other epithelial organs such as the liver, lung, kidney, pancreas, gastrointestinal tract, breast, milk glands, and even the cancers which are induced by these tissues ^16^. CK19 has the highest frequency and is useful in the diagnosis of CTCs and a variety of epithelial-derived diseases ^17^. Furthermore, this marker was successfully used as a very sensitive and superior marker for early diagnosis and development of breast cancer in tumor cells in bone marrow, axillary lymph nodes, and peripheral blood ^18^. CK8 marker can be used along with CK7, CK18, and CK19 markers to detect CTCs ^16^. EpCAM is another epithelial marker for diagnosis. It is a trans-membrane glycoprotein^19^ which expresses in a variety of epithelial cancers such as breast, stomach, prostate, and esophagus ^20^. This marker has high expression in primary and metastatic breast cancer about 100 to 1000 times higher than the healthy mammary breast cells. Like the CK7, CK8, CK18, and CK19, EpCAM is also used for diagnosis of CTCs ^21^. CTCs are known for early detection of potential metastasis, determining and monitoring the efficacy of individualized treatment regimens.

Also, immunomagnetic flow cytometry is considered as one of the essential methods for CTCs diagnosis. In this way, the enrichment process is usually one of the prerequisites for any diagnosis and separation protocol. CTC is diagnosed during two stages in this technique; the CTCs enrichment is the first stage, and the CTC diagnosis is the second stage ^22^. The enrichment stage includes the interaction of target cells with conjugated antibodies to immunomagnetic granules. There are several enrichment methods for CTCs, such as separation with immunomagnetic pellets based on density, centrifugation, and size. Each enrichment method has positive and negative selection processes ^22,23^. A group of markers are used for the diagnosis of enriched CTCs, and ultimately, the tumor cells were examined by flow cytometry ^6^. In this study, immunomagnetic flow cytometry with CK7, CK8, CK18, and CK19 (Pan-C) antibodies and EpCAM microbeads were used to detect CTCs in breast cancer and they were compared with flow cytometry. Finally, specificity and sensitivity of two methods by CK19 flow cytometry and immunomagnetic separation was also evaluated for CTCs detection in the blood.

## Materials and Methods

Patients referred to Milad Hospital were selected in a non-randomized study after diagnosis before any treatment. The cohort included 35 patients and 35 healthy women. The healthy subjects voluntarily participated in this study after medical examination. Age-matched female patients and healthy subjects were in the same age groups, between 22 and 74 years. The inclusion criteria were as follows; patients with breast cancer diagnosis before onset of the treatment process were selected and the written informed consent was obtained. Exclusion criterion was the secondary primary malignancy. The informed consent was obtained from all patients for using their blood samples. The samples were collected using protocols approved by the review board. The samples were transferred to the Pasteur Institute of Iran and used in this study. This study was approved by the National Ethical Committee of the Pasteur Institute of Iran (Ethical approval No. 4552). All methods were performed in accordance with the relevant guidelines and regulations based on ethics committee.

Breast cancer diagnosed patients were at I–IV stages of the disease. The completed questionnaires contained information on patient’s age, marital status, family history of cancer, and then peripheral blood was taken from healthy cases and patients.

### Blood sample preparation

First, 5 *ml* of blood was collected from patients and healthy samples. Human white blood cells were isolated from adult peripheral blood using RBC lysis buffer Magnetic Activated Cell Sorting (MACS). Briefly, 1 *ml* blood and 5 *ml* RBC lysis buffer were mixed with vortex and kept on ice for 15 *min*, then were centrifuged (1500 *rpm* for 10 *min*). Cells were suspended in 5 *ml* RBC lysis buffer and centrifuged followed by twice washing with Phosphate-Buffered Saline (PBS).

### Cell culture

Human breast (T47D) and cervical (HeLa) cancer cell line were obtained from the cell bank of Pasteur Institute of Iran. Human cervical (HeLa) cancer cell line was used as a negative breast cancer control. Briefly, cell lines were cultured in RPMI 1640 (Gibco) supplemented with 10% fetal bovine serum (FBS) (Gibco, Invitrogen) and 700 *μl* penicillin-streptomycin (Biosera). Cells were kept at 37°*C* in a humidified CO_2_ incubator (5% CO_2_). Then, cell lines grown in monolayer were harvested by washing the plates once with PBS, pH=7.3, and then the cells were incubated with trypsin/EDTA (Biosera) for 2–5 *min* at 37°*C*. Finally, cells were counted using hemocytometer.

### Flow cytometry

In this study, blood cells were fixed with 4% paraformaldehyde (Merck) in PBS 1× for 20 *min* at room temperature. Then, cells were washed twice with 1% PBS/FBS and were permeabilized with ice-cold 100% methanol (Merck) for 30 *min* at 4°*C*. Then, cells were washed twice with 1% PBS/FBS and were blocked and fixed with 1% Bovine Serum Albumin (BSA) for 45 *min* at room temperature. Then, cells were stained with the FITC-conjugated mouse anti-human CK19 antibody (Abcam, diluted 1:300 in PBS 1×) or FITC-mouse IgG2a isotype antibody (Abcam, diluted 1:300 in PBS 1×) as a negative control and incubated for 1 *hr* at room temperature. After twice washing with 1% PBS/FBS, detection of bound antibodies was determined by flow cytometry (Cyflow), and the results were analyzed with the following program.

### Immunomagnetic flow cytometry

Blood sample (1 *ml*) was added to 5 *ml* of RBC lysis buffer. The sample was suspended and incubated on ice for 10 *min*. Next, 6 *ml* of PBS 1× was added to the sample, and then centrifuged (1500 *rpm* for 10 *min*). Then, 1 *ml* of PBS 1X was added to the pellet sample and centrifuged (1500 *rpm* at 4°*C* in 10 *min*). Afterward, the supernatant was discarded and MACS cell separation kit (Miltenyi Biotec GmbH, Bergisch Gladbach, Germany), or magnetic activated cell sorting captured cells by labeling with immunomagnetic microbeads. Permeabilization and fixation step was done by a membrane or intracellular staining. Magnetic beads were linked to anti-epithelial antibodies for positive selection through EpCAM. The procedure was applied according to the manufacturer’s instructions. AutoMACS rinsing buffer (1 *ml*) was added to the sample and centrifuged (1500 *rpm* at 20°*C* for 10 *min*). The supernatant was discarded and then 300 *μl* of autoMACS rinsing buffer and 50 *ml* of blocking reagent were added. EpCAM MicroBeads ® (MACS) (50 *ml*) were added, so the cells were enriched with EpCAM microbeads, and then the sample was incubated at 4–8°*C* for 30 *min*. Columns were placed on MiniMACS separation, and then Macs Column was washed with 500 *ml* of autoMACS rinsing buffer. The sample with EpCAM microbeads was transferred to Macs Column, and 1 *ml* of rinsing buffer from autoMACS was added. AutoMACS (500 *ml*) rinsing buffer was added to Macs Column, and then MiniMACS was separated from Macs Column, and its solution was discarded. Inside fix buffer (500 *μl*) was added to the sample and incubated at room temperature for 20 *min*. The inside perm (500 *μl*) and 50 *μl* of anti-cytokeratin (Pan-CK) antibody were mixed and incubated at room temperature for 10 *min*. Then, 50 *μl* of Anti-IgG conjugate PE (Phycoerythrin) was added to the sample and incubated at room temperature for 20 *min*. Then, flow cytometry analysis was done by control samples with no anti-cytokeratin. EpCAM microbeads were also considered as the negative control in this experiment.

### Sensitivity and specificity of two methods

Sensitivity of medical diagnosis test is the ability of a test to correctly identify those with positive rate, whereas specificity is the ability of the test to identify those without the disease, with correctly negative rate. T47D cells were serially diluted with human peripheral blood leukocytes and stained with the CK19 biomarker. A total of 70 blood samples, including 35 healthy and 35 patients with breast cancer, were tested by flow cytometry to quantify the CK19 expression for specificity. Briefly, 1 *ml* of blood and 5 *ml* of RBC lysis buffer were mixed and kept on ice for 15 *min*. Subsequently, the cells were fixed with 4% paraformaldehyde for 20 *min* at room temperature. They were permeabilized with methanol for 30 *min* on ice. Then, the cells were incubated with FITC conjugated CK19 antibody or FITC-mouse IgG2a isotype antibody as the negative control for sensitivity.

In a ROC curve, the true positive rate (sensitivity) is plotted in function of the false positive rate (specificity) for different cut-off points of these two parameters. The area under the ROC curve was measured to see how well a parameter can distinguish between two diagnostic groups (Diseased versus normal).

### Statistical analysis

Chi-square test was used to analyze biomarkers expression in peripheral blood of patients before clinical treatment. Pearson chi-square was performed to compare biomarkers expression level in peripheral blood between patients at stages I–IV. p-values of less than 0.05 were considered statistically significant.

## Results

### Patient characteristics

The characteristics of 35 patients enrolled in this study are listed in table 1. The age range of patients was from 22 to74 years old, and the median age was 50 years old (Table 1). There were 33 patients with no evidence of metastasis and two patients with metastatic breast cancer.

**Table 1.** Pathological characteristic of patients

**Tumor size**	**Number (%)**
< 1 *cm*	7 (20)
1–2 *cm*	23 (65.7)
> 2 *cm*	5 (14.3)
**Clinical stage**	
I	4 (11.43)
II	22 (62.85)
III	6 (17.15)
IV	3 (8.57)
**Clinical grade**	
G1	3 (8.57)
G2	17 (48.57)
G3	11 (31.43)
ND	4 (11.43)
**Lymph node**	
N0	14 (40)
N1	16 (45.71)
N2	1 (2.86)
N3	1 (2.86)
NX	3 (8.57)
**Histology**	
Invasive ductal carcinoma	31 (88.58)
Invasive lobular carcinoma	2 (5.71)
Other types of breast cancer	2 (5.71)
**Age**	
<50 years	18 (51.42)
≥50 years	17 (48.58)
**Molecular markers**	
**ER**	
Positive	20 (57.14)
Negative	8 (22.86)
ND	7 (20)
**PR**	
Positive	18 (51.43)
Negative	10 (28.57)
ND	7 (20)
**P53**	
Positive	11 (31.43)
Negative	14 (40)
ND	10 (28.57)
**Her 2**	
Positive	12 (34.3)
Negative	16 (45.7)
ND	7 (20)
**Ki 67**	
Positive	21 (60)
Negative	2 (5.7)
ND	12 (34.3)
**Distant metastasis**	
Metastasis	2 (5.7)
Without metastasis	33 (94.3)

Not defined (ND).

Clinical assessment was based on histological reports at different stages. Our study population contained patients at different stages. Breast cancer staging (I–IV) was classified according to the standard criteria based on data of TNM (Tumor, Nodes and Metastases) and American Joint Committee on Cancer Staging System (AJCC). The tumors were histologically graded according to the modified Bloom-Richardson grading system. Clinically classified information concerning age, diagnosis and clinical pathology of breast cancer patients is shown in table 1.

### Flow cytometry

**Figure 1.**
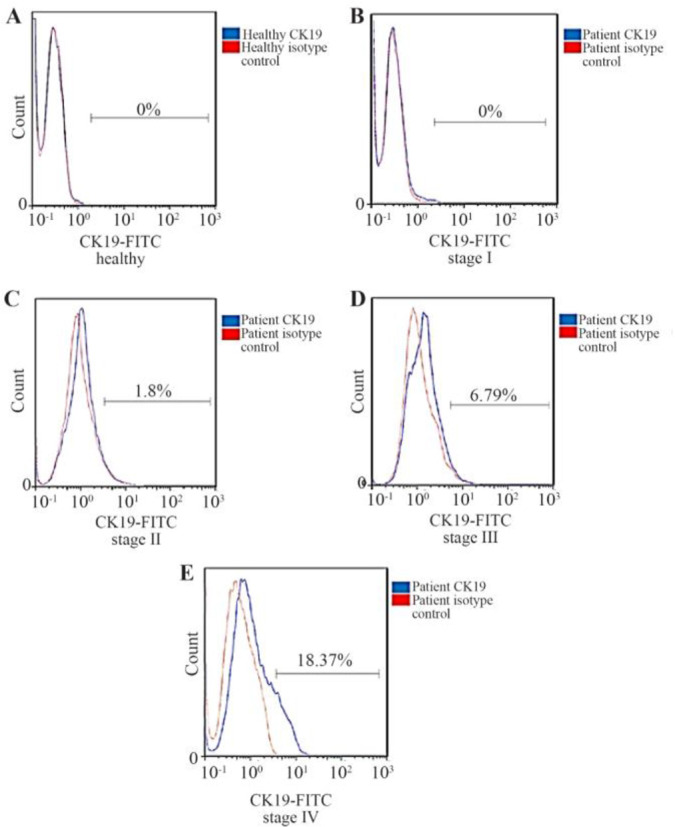
Flow cytometry diagrams of blood cells (%) stained with the FITC-conjugated mouse anti-human CK19 antibody (Abcam) or FITC-mouse IgG2a isotype antibody (Abcam) as the negative control, in (A) healthy subjects (0%), and patients at (B) stage I (0%), (C) stage II (1.8%), (D) stage III (6.79%), and (E) stage IV(18.37%).

Samples were stained with FITC-conjugated mouse anti-human CK19 (Abcam) and FITC-mouse IgG2a isotype antibody (Abcam) as a negative control by flow cytometry (Figure 1). Healthy subjects and patients at stage I, stage II, stage III, and stage IV were detected.

**Figure 2.**
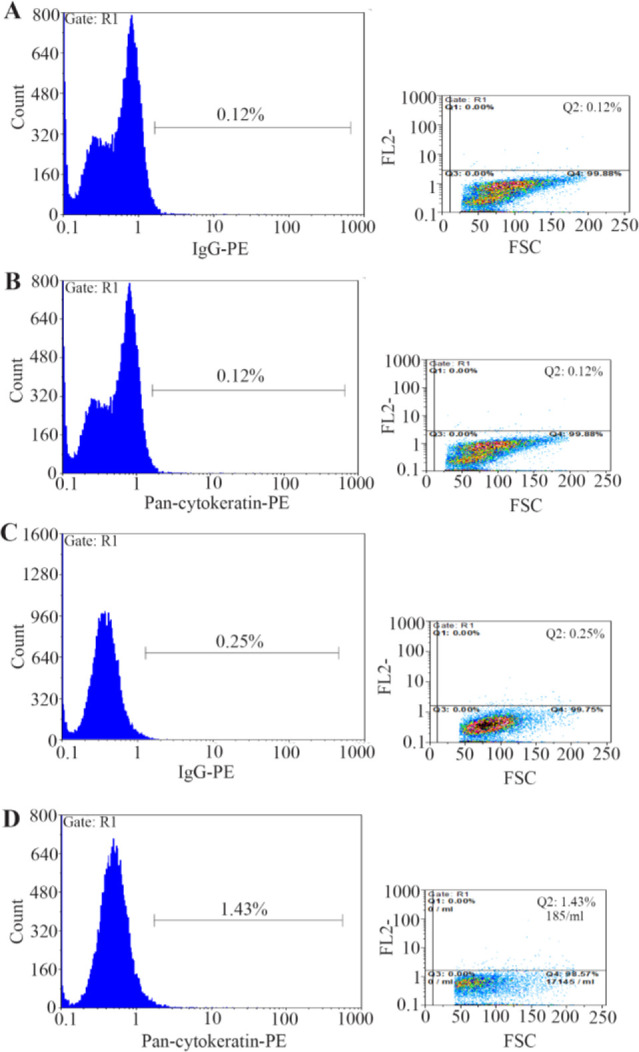
Flow cytometry analysis of blood cells from: A) Healthy subjects (0.1% of cells detected with control antibody). B) Patients at stage I (0.1% of cells detected with Pan-antibody). C) Patients at stages II–IV (0.25% of cells detected with control antibody). D) patients at stages II–IV (1.43% of cells detected with Pan-antibody).

Twenty patients (57.14%) were found to have CK19^+^ cells in peripheral blood, 12 samples were CK19 positive at stage II, five samples at stage III and two samples with breast cancer at stage IV but CK19 was not detected in patients at stage I and healthy controls by flow cytometry. In this study, magnetic flow cytometry showed CTC with Pan-CK markers expression. Firstly, immunomagnetic flow cytometry was utilized by EpCAM magnetic beads, and secondly, CK7, CK8, CK18, and CK19 (Pan-CK) biomarkers were used for CTC detection. The positive and negative samples were determined, and the results are presented in table 2. CTC in peripheral blood of 14 patients (40%) was detected; six samples were CTC positive at stage II, six samples at stage III and two samples with breast cancer at stage IV while CTC was not detected in patients at stage I and healthy controls by immunomagnetic flow cytometry. Flow cytometry analysis showed histograms and dot diagram. In this method, also biomarkers in Pan-CK were not expressed in healthy blood samples and patient’s blood samples at stage I of disease (Figures 2A and B). The expression of these biomarkers was also evaluated in blood samples at different stages of disease (Figures 2C and D). The CK19 gene expression was statistically significant (p=0.037) in flow cytometry. By immunomagnetic flow cytometry, CTC detection was statistically significant (p=0.001). In table 2, CTCs detection at different stages was compared in two flow cytometry by CK19 and Pan-CK antibody.

**Table 2.** CTCs detection at different stages of breast cancer

**Samples**	**Total number**	**Flow cytometry**	**Immunomagnetic flow cytometry**

		**CK19+ (%)**	**EpCAM/CK7, CK8, CK18, CK19 (Pan marker)+(%)**
**Healthy control**	35	0	0
**Stage I**	5	0	0
**Stage II**	22	12 (54.5)	6 (27.2)
**Stage III**	6	5 (83.3)	6 (100)
**Stage IV**	2	2 (100)	2 (100)
**Total patients**	35	20 (57.14)	14 (40)

### The specificity and sensitivity of flow cytometry

**Figure 3.**
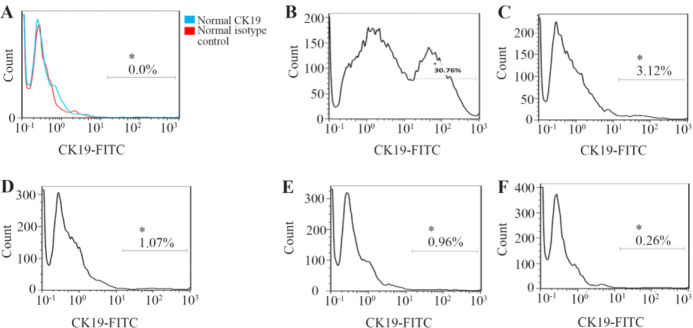
CK19 expression in human breast cancer cell line (T47D) diluted with human white blood cells in different dilutions. Healthy adult white blood cells (A) T47D cells mixed with healthy adult white blood cells in different dilutions of 1:1 (B), 1:10 (C), 1:10^2^ (D), 1:10^3^ (E), and 1:10^4^ (F). The cell mixture was stained with FITC-anti-CK19 antibody for detection of the CK19 expression.

Flow cytometric analysis indicated that human breast cancer cell line (T47D) expressed the high level of CK19 biomarker (Figure 3). However, healthy peripheral white blood cells had no CK19 expression (Figure 3).

#### Sensitivity:

T47D cells (1.5×10^6^) were mixed with healthy white blood cells at different ratios of 1:1, 1:10, 1:10^2^, 1:10^3^, and 1:10^4^ to determine the sensitivity of flow cytometry. CK19+ cells were detected by flow cytometry, and they were consistent with the ratios of T47D and white blood cells. It was demonstrated that flow cytometry could distinguish the percentage of CK19 expressing cells, even one T47D cell in 10^4^ white blood cells.

#### Specificity:

The specificity and sensitivity of flow cytometry methods by Anti-CK19 and Pan-CK antibody with EpCAM magnetic beads was also calculated at different stages (Table 3).

**Table 3.** The specificity and sensitivity of flow cytometry methods

	**Flow cytometry by Anti-CK19**	**Immunomagnetic flow cytometry (MACS) CK7, CK8, CK18, CK19, EpCAM (Pan marker)**	**Total number**	**p**
**Healthy control**	0	0	35 (100%)	-
**Stage I patients**	0	0	4	-
**Stage II patients**	12 (54.5%)	6 (27.2%)	22	-
**Stage III patients**	5 (83.3%)	6 (100%)	6	-
**Stage IV patients**	2 (100%)	2 (100%)	2	-
**Not defined**	1 (100%)	0	1	-
**Total patients**	20 (57.1%)	14 (40%)	35 (100%)	-
**Specificity**	100%	100%	100%	0.085^*^
**Sensitivity**	57.1%	40%	97.1%	**0.318**
**p-value at different stages**	0.037^*^	0.001^*^	-	-

* Statistically significant p-value.

**Figure 4.**
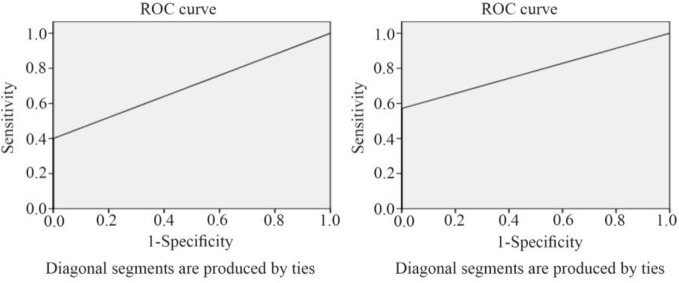
ROC curve drawing based on sensitivity and specificity of Anti-CK19 flow cytometry and immunomagnetic combing flow cytometry methods.

The ROC curve is created by plotting the exact Positive Rate (TPR) against the False Positive Rate (FPR) at various threshold settings. The area under the ROC curve was measured accurately. The flow cytometry by using anti-CK19 has shown more area under the ROC curve in figure 4.

## Discussion

Breast cancer is one of the most common forms of cancer which causes death every year. It is estimated that one out of eight women develops breast cancer ^3^. According to Iran’s cancer registration, breast cancer is in the first rank cancers among women ^3^. Despite the advances in the early diagnosis and treatment of breast cancer, it is still the leading cause of death among women, so there is still a need for new strategies to assess breast cancer ^24^.

In recent years, the CTC was considered a unique target for understanding the disease development, prognosis, and treatment of breast cancer ^9^. In the last decade, CTC detection methods and their clinical utility were studied. CTCs include information on genetic and epigenetic profiles associated with cancer development, progression, and response to therapy that comes from circulating tumor DNA (ctDNA) in the blood of cancer patients ^25,26^.

On the other hand, as an early detection, biomarker ctDNA holds promise in cancers for which there are currently no accepted screening methodologies, such as ovarian, pancreatic, and gastric cancers. As an early cancer detection biomarker, ctDNA testing may be synergistically used with other multi-omic biomarkers to enhance early detection. However, some challenges including accuracy of early stage diagnosis of disease, and high costs of early cancer screening tests need to be addressed ^27^.

CTC levels were measured in the blood of patients by the CellSearch system with breast cancer. The Cell-Search system is the only FDA approved technology for CTC detection in cancer patient management ^28^. Despite applying novel techniques, any CTC-based test has only been introduced into clinical use and has not been implemented into routine clinical practice. There is much evidence that the presence of CTCs is associated with disease development in patients with breast cancer. The diagnosis of CTCs in patients’ blood with breast cancer is one of the most essential goals in clinical trials because there are a few number of these cells in the blood at the early stages of the disease ^29^. Also, it has been shown that these cells could reflect cancer development ^30^. Therefore, the diagnosis of CTCs could be useful for cancer diagnosis as well as prediction and treatment monitoring ^31^. There are different CTCs detection methods, and most of them are based on biomarker detection by flow cytometry ^22,32^. These few techniques for tumor cell determination in blood have advantages and disadvantages. The flow cytometry is a conventional method which can simultaneously examine multiple parameters and quickly analyze cells ^22^. This approach has higher and more specific specificity than other detection methods. The immunomagnetic method is based on enrichment with magnetic beads. This technique maintains cell integrity and is an easy way to diagnose tumor cells ^22^. Individual markers are used for diagnosing CTCs in breast cancer, and they have high expression in epithelial cells ^12^. CKs are among the epithelial markers which have been converted to a standard marker for diagnosis of CTCs ^31^. CK19 has a maximum frequency in the diagnosis of CTCs and is proposed to be a useful marker in cancers with epithelial origin ^17^. In addition to CK19, other epithelial markers such as EpCAM, Her-2, MUC-1 and mammaglobin and a set of CKs namely CK8, CK18, and CK19 and in some cases (Pan-CK) CK7, CK8, CK18, and CK19 were reported to be used for CTC diagnosis ^33–35^. CELLSEARCH was approved by FDA and EpCAM^+^ CTCs were immunomagnetically enriched, and then the fluorescently labeled antibodies were analyzed. A significant limitation of this method is reagents, and CELLSEARCH laboratory equipment ^36^.

In this study, 35 blood samples from patients at various stages of breast cancer disease (I–IV) and 35 healthy blood samples were used as controls to diagnose the CTCs in the blood of subjects. The immunomagnetic flow cytometry by Pan-CKs antibody was applied for CTCs detection in blood. It should be noted that the blood samples were taken from patients before any treatment including chemotherapy, radiotherapy or hormone therapy because it is likely that the changes will be made in cells during the treatment and will lead to false expression or lack of its gene expression. On the pathological characteristics of patients, the study analyzed factors such as age, stage of disease, tumor size, type of tumor, lymph node involvement and molecular ER, PR, Ki67, P53, and Her-2 markers expression. In this study, it was shown that there is a significant correlation between Pan-CK expression and stage of disease with p*<*0.05. Furthermore, biomarker expression was independent of lymph node involvement, age, tumor size, type of tumor, and molecular ER, PR, Ki67, P53, and Her-2 markers expression in cancerous tissue. The results of this research indicated that CK19 marker presents an appropriate and independent marker at different stages of disease for CTCs detection in breast cancer patients. The expression of several markers, including CK19 has been shown in other studies of breast cancer. It was shown that the CK19 expression was significantly different between patients and healthy controls ^37,38^. In this study, the blood samples from Iranian women with breast cancer were studied to diagnose the CTCs by immunomagnetic flow cytometry. In this method, the CTCs were detected from 40% of patients with Pan-CK. Moreover, the statistical analysis indicates that the expression of these markers was significant at different stages of disease (p<0.05). Benefits and drawbacks of flow cytometry and CELL-SEARCH assays already showed that epithelial tumor cells detection by CELLSEARCH is comparable with flow cytometry, and both methods have the same sensitivity and specificity ^36^.

In this study, statistical analysis was performed to find out the correlation between pathological characteristics (Table 1) of patients such as temperature, age, disease, stage, tumor size, type of tumor, lymph node involvement, and molecular ER, PR, Ki67, P53, and Her-2 markers. There is a significant correlation between biomarker expression and stage of disease with p<0.05. Furthermore, it was demonstrated that the presence of CTC at different stages of breast cancer is independent of lymph node involvement, tumor stage, size, and situation of ER and PR receptors.

## Conclusion

In conclusion, CK19 biomarker detection in peripheral blood can widely be used for breast cancer detection and therapeutic monitoring in patients. Also, flow cytometry seems to be the most specific and feasible method to monitor CTCs as a prognostic or predictive indicator in breast cancer patients.
